# Book Reviews

**Published:** 1895-03

**Authors:** 


### *BOOK REVIEWS.*

Diseases of the Ear: A Text-Book for Practitioners and Students of Medicine. By E. B. Dench, Ph.B., M.D., Professor of Diseases of the Ear in Bellevue Hospital Medical College, N. Y. D. Appleton & Co.

One has only to read this volume in order to see its worth. Whether there was need at present for a new text-book on Otology must be seen from the success which will be met with by this work of Dr. Dench. However, we have no hesitancy in saying that it is the best work of its kind by an American author. Dr. Dench is, perhaps, one of the leading exponents of intra-tympanic surgery, and while his views upon this subject are, perhaps, more radical than the majority of aural surgeons, yet they must be thoughtfully considered, coming as they do from one who is so well and favorably known. It is almost impossible to display any originality in writing a work upon the ear, yet in this text-book the author has dealt in no superficial vagaries, but he speaks as one with a large amount of clinical experience, and thus gives to the reader those points which are of practical importance. In chapters I. and II. the author treats of the anatomy and physiology of the ear, and while in these chapters there is no room for originality, yet the subject is unusually clear and distinctly set forth, made still more so by plates which are drawings from the author's own specimens. Two plates especially must be noticed, one giving the arterial and the other the venous supply to the ear, which in other works upon the same subject has not been so forcibly presented.

Chapter III. on physical examination is unusually clear and practical. One sentence in this chapter accords so evenly with my own views that I wish to quote it here: "There can be no question as to the value of Politzer's method both as a diagnostic and therapeutic procedure, but its use should, I think, be restricted to certain cases, and it should not be adopted to the exclusion of catheterization of the tube." The author devotes some little space, and rightly so, to the subject of auscultation of the Eustachian tube and tympanic cavity, which is indeed an important procedure, and one which has not been dwelt upon by other authors to an extent which the subject demands.

In regard to the surgical treatment of deafness and chronic otorrhea, the author dwells at considerable length, and while a great many of us may not be as enthusiastic an advocate as the author himself, it is certainly an important and brilliant field for further investigation.

Taking the work as a whole, it is a most excellent text-book, and one which will no doubt become standard as a safe book for the general practitioner and aurist to follow. D. R.

---

RELATIONS OF DISEASES OF THE EYE TO GENERAL DISEASES. By Max Knies, Professor Extraordinary at the University of Freiburg. Edited by Henry D. Noyes, A.M., M.D., Professor of Ophthalmology in Bellevue Hospital Medical College, N. Y. William Wood & Co., Publishers.

This work by Professor Knies forms a supplementary volume to every manual and text-book of practical medicine and ophthalmology. The gist of the book and the full intent of its author can be readily seen by quoting the first paragraph of his preface:

"Diseases of the eye often possess important significance in relation to the diagnosis and correct understanding of diseases of other organs; and as both the physician and the ophthalmologist should comprehend the mutual relations of such affections both in diagnosis and treatment, I have been led to add to my text-book on diseases of the eye, a second part to cover the relations of the eye and its diseases to diseases of the rest of the body."

Since there is such a close relationship between the eye and our nervous system, it is quite natural that this latter should take a prominent place in various morbid states of our visual organ. Hence we find that the first half of the book discusses this relationship of the eye and the nervous system. The visual centers and other centers in the brain and spinal chord are minutely discussed, thus affording both the ophthalmologist and physician a clearer understanding of the relationship between these organs.

Chapter II. treats of the relationship of skin diseases with those of the eye, while in chapter III. we are brought to consider that of the digestive organs.

In chapter IV. the diseases of the respiratory organs in their relationship are treated, and though by no means given that importance which, according to our ideas, are justly due, yet there are many points of practical importance. The importance of nasal disorders and their proper treatment has by no means received that attention by European specialists that it has in America.

Chapters V., VI. and VII. treat respectively of the relationship of diseases of the circulatory organs and diseases of the urinary and sexual organs.

The last chapter, which is the most important save the first, treats of poisons and infectious diseases. I say important, because this relationship is now well recognized.

The volume deals with an important subject and will be found as a most ready book for reference in any physician's library. D. R.

---

TWENTIETH CENTURY PRACTICE: AN ENCYCLOPEDIA OF MODERN MEDICAL SCIENCE BY LEADING AUTHORITIES OF EUROPE AND AMERICA. Edited by Thomas L. Stedman, M.D., New York. Published by William Wood & Company, New York City.

This is the first of twenty volumes which will be issued, one every three months, until the series is complete. It will be an immense undertaking in medical book-making, but if we are to judge by the long list of able teachers and clinicians who will contribute to the enterprise, we will have in the end the most valuable series of reference-books ever offered to the profession.

The first volume refers to "Diseases of the Uropoietic System." Diseases of the kidney are described by Dr. Francis Delafield, of New York. No one could do it better. Dr. Delafield's description of these diseases is the clearest and simplest that we have ever read. Several excellent engravings (taken from photographs) illustrate this part of the volume. The next section is a description of the surgical diseases of the kidneys and ureters, by Mr. Reginald Harrison, of London. Diseases of the bladder make up another section, also by the last-named author. Dr. G. Frank Lydston, of Chicago, describes the diseases of the prostate and male urethra. Some of the author's views in this section may be unfavorably criticised, but he presents them with force and conviction apparently born of experience. Diseases of the urine, hematuria, accidental albuminuria, phosphaturia, etc., (except diabetes), are described by Mr. Hurry Fenwick, of London. The volume closes with a section on diseases of the female bladder and urethra by Dr. Howard A. Kelly, of Baltimore. In this section will be found some matter (we believe) not published before, relating to the endoscopic inspection of the female bladder and the catheterization of the ureters.

The entire volume is carefully prepared, and all subjects described with more attention to thoroughness and detail than can be given in smaller works. The book is a series of exhaustive monographs which will be standard and authoritative for many years to come. The set of twenty volumes will be a whole medical library.

---

LIPPINCOTT'S MAGAZINE FOR MARCH, 1895.

The complete novel in the March issue of *Lippincott's* is "A Tame Surrender," by Captain Charles King. Departing from this author's usual field, the purely military, it deals with the Chicago strike, the riots and their suppression, and the loves of a United States lieutenant and a high-minded young lady who works a typewriter. It is her "tame surrender," after long resistance, which gives the tale its title.

The other stories, all very short, are "Fulfilment," by Elizabeth Knowlton Carter, "The Luck of the Atkinses," by Margaret B. Yeates, and "One of the Wanted," by B. B.

Two brief scientific articles are supplied by George J. Varney, "Electric Locomotives on Steam Roads," and "The Story of the Gravels," by Harvey B. Bashore.

"A Glimpse of Cuba," by James Knapp Reeve, is a vivid and readable sketch. Isabel F. Hapgood writes of "Furs in Russia," and W. D. McCracken on "A Question of Costume."

Professor William Cranston Lawton discusses "The Artist's Compensations," Professor H. H. Boyeson furnishes "A Youthful Reminiscence," and C. W. Lucas, as "Doolittle," writes "An Open Letter" to Mrs Grundy.

The poetry of the number is by Professor Charles G. D. Roberts and Richard Burton.

